# High-Throughput Quantification of 32 Bioactive Antioxidant Phenolic Compounds in Grapes, Wines and Vinification Byproducts by LC–MS/MS

**DOI:** 10.3390/antiox10081174

**Published:** 2021-07-23

**Authors:** Eleni D. Myrtsi, Sofia D. Koulocheri, Vassilios Iliopoulos, Serkos A. Haroutounian

**Affiliations:** Laboratory of Nutritional Physiology and Feeding, Department of Animal Science, School of Animal Biosciences, Agricultural University of Athens, Iera Odos 75, 11855 Athens, Greece; elenamirtsi@aua.gr (E.D.M.); skoul@aua.gr (S.D.K.); heliopoylos@hotmail.com (V.I.)

**Keywords:** phenolic antioxidants, polyphenols quantitation, grape stems, grape pomace, wines, LC–MS/MS

## Abstract

The well-established, health-benefitting effects of grapevines and derivatives (wines and vinification byproducts) are attributed to their antioxidant phenolic content. The dearth of an efficient method for the simultaneous quantitation of antioxidant phenolics prompted us to develop a novel method utilizing triple quadrupole LC–MS/MS for the accurate, fast, simultaneous quantitation of the 32 most abundant grapevine phenolics. The fully validated, novel method is capable to simultaneously record the quantitative presence of 12 phenolic acids, 19 polyphenols and coniferyl aldehyde (a phenolic compound extracted from cork stoppers into wines) and is applicable for the determination of antioxidant phenolics content of grape berries, pomace, stems and wines. Its utility was demonstrated for three native Greek grapevine varieties, two red (Mandilaria and Aidani mavro) and one white (Monemvassia). Results herein highlighted the stems of the Monemvassia white variety as particularly rich in antioxidant phenolics such as the flavonol monomer (+)-catechin (387 mg/kg) and the dimer procyanidin B1 (400 mg/kg) along with stilbene phytoalexin *trans*-resveratrol (24 mg/kg). These results are in line with the TPC, TFC and TTC content of stems and the determined antioxidant capacities, highlighting the stems of this *Vitis vinifera* variety as potentially exploitable source of antioxidant phenolics.

## 1. Introduction

The grapevine is a thermophilic plant cultivated in the temperate zone around the globe. According to Food and Agriculture Organization (FAO) data, 71% of world grapevine production is used for winemaking. The global production of wine for the year 2018 exceeded 29 Mtones, highlighting wine as one of the most important agricultural products for international trade. The vast amount of global wine production is located in Europe (62%, 18 Mtones), mainly in Italy, France and Spain [1].

Grapes contain large amounts of phytochemicals that provide to the respective wines their sensory characteristics of color, aroma and astringency [2] as well as various properties beneficial to health [3,4,5]. Among these phytochemicals, antioxidant phenolics constitute the the most prominent and well-studied class of compounds [6,7]. They consist of polyphenols, defined as the compounds that possess multiple aromatic rings with one or more hydroxy groups (flavonoids, anthocyanins, tannins, stilbenes) and phenolic acids, which are compounds containing a carboxy-substituted aromatic ring with one or more hydroxyl groups attached [6,8]. Functionally, the antioxidant phenolic compounds are classified into secondary metabolites, a class of plant-derived compounds with diverse chemical structures and functions, which are produced during the physiological growth of grapevines or as their response to various forms of environmental stress [8,9,10]. Their presence in grapevines is associated with the quality and authenticity of wines [11,12,13] along with a broad spectrum of bioactivities including antimicrobial [14,15], anticancer [16,17,18], antiaging [19], antiglycogenic [20] and cardiovascular system-protective [21,22] properties. During the last three decades, the biological activities of grapevine phenolic compounds have been extensively exploited, providing valuable information that has attributed their health-benefitting effects to their strong antioxidant properties. Thus, it has been established that these compounds act as potent free radical scavengers, electron or hydrogen donors and strong metal chelators, which prevent the development of diverse harmful events such as lipid peroxidation [23], DNA damage [24], etc. Additionally, grape phenolics display the capability to provide protection from the ROS-induced DNA damage of various cancer cells (e.g., liver and cervical) [25] or the oxidative damage of cell constituents, limiting the risk of developing chronic neurodegenerative conditions associated with oxidative stress such as dementia and Parkinson’s and Alzheimer’s diseases [3,5]. Finally, it is noteworthy that several studies have indicated that the incorporation of grape phenolics into a daily diet limits the possibility of developing cancer, cardiovascular diseases, neurodegenerative diseases, diabetes and osteoporosis [26].

The above-mentioned health-benefitting effects of grapevine phenolics have initiated a sharply growing market for their utilization as active ingredients by the cosmetic, pharmaceutical [27] and food supplement industries [28,29]. Thus, vigorous research activity has been initiated toward the identification and efficient recovery of various antioxidant phenolics from vinification by-products, as pure compounds and/or enriched extracts [17,30]. Consequently, there are many scientific reports concerning the phenolic compound content of different Vitis vinifera varieties [31,32], the most widely cultivated grapevine species, along with various methods for the determination of their abundancy [32,33,34,35] and/or recovery procedures [14,36,37]. Thus, there is an emerging need for the development and validation of a fast and accurate method, which will be applicable for samples obtained from grapes, wines and vinification by-products (grape pomace and stems), for the simultaneous determination of a large number of bioactive phenolics. It must be noted however, that the diversity and structural complexity of grape phenolics constitute serious drawbacks for the development of an efficient, universal analytical method for simultaneous assessment from diverse samples. Most of the methods developed to date concern applications of reversed-phase HPLC, with the disadvantages of long durations of run-times and the limited number of simultaneously determined phenolics [35,38].

The study herein refers to the development, validation and application of a high-throughput method for the simultaneous determination of the quantitative presence of the 32 most common antioxidant phenolic compounds found in grapevines. The method was developed using reverse-phase, high-pressure liquid chromatography coupled with tandem MS/MS, which allowed the fast and highly sensitive simultaneous determination of the antioxidant phenolics found in grapes, wines and vinification byproducts. In the context of the present work, three native Greek *Vitis vinfera* grapevine varieties, cultivated on the island of Paros, were studied, along with their respective wines (white, red and rose) and vinification byproducts (pomace and stems). Moreover, the antioxidant phenolics of these products were identified and quantified along with their contents of total phenolics (TPC), flavonoids (TFC) and tannins (TTC) as well as the estimation of their antioxidant capacities through the performance of DPPH and FRAP assays.

## 2. Materials and Methods

### 2.1. Grapes, Wines and Vinification Byproducts

All samples studied were provided by the Moraitis Winery, located on the island of Paros, Greece. The fresh grape berries belonging to native red (Mandilaria, Aidani mavro) and white (Monemvassia) varieties of the *Vitis vinifera* species were harvested at their commercial maturity from the winery’s vineyard during the summer of 2020. Grape berries were manually destemmed and unseeded, weighed, freeze dried, mill powdered and stored in a freezer. The vinification byproducts (grape pomace and stems) were separated after the 2020 production process, air dried in a dark room, mill powdered and stored at room temperature until processing. The studied wines were provided by the winery as red (Mandilaria), white (Monemvassia) and rose (Aidani mavro) wine samples.

### 2.2. Chemicals and Standards

All solvents used for the extractions of phenolic compounds from grapevines and vinification byproducts were purchased from Carlo Erba and Fisher Chemicals as analytical-grade solvents. Solvents used for the LC–MS/MS determinations were obtained as LC–MS grade from J.T. Baker (water and acetonitrile) and Fisher Chemicals (formic acid).

All standards used for the assessments of phenolic compounds were obtained from Sigma-Aldrich except epigallocatechin gallate and quercetin-3-*β*-D-glucoside, which were provided by Extra Synthese; coutaric, fertaric and caftaric acids, which were obtained from Phytolab; and ferulic acid, bought from Fluka. Folin–Ciocalteu’s reagent, 2,2-diphenyl-1-picrylhydrazyl (DPPH), and 2,4,6-tris(2-pyridyl)-s-triazine (TPTZ) were purchased from Sigma-Aldrich, while 6-hydroxy-2,5,7,8-tetramethylchroman-2-carboxylic acid (Trolox) and vanillin were obtained from Acros Organics. Anhydrous sodium carbonate, sulfuric acid 98% and hydrochloric acid were purchased from Chem-Lab; glacial acetic acid from Sigma-Aldrich; hexahydrate aluminum chloride from Fluka; and hydrate sodium acetate from Merck. Hexahydrate ferric chloride and heptahydrate iron sulfate were obtained from Alfa Aesar.

### 2.3. Sample Preparation

The extraction of phenolics from solid samples (grapes, vinification byproducts) was implemented as follows: 50 g of dried, powdered sample were added into a 200 mL mixture of MeOH/H_2_O/1.0 N HCl (90:9.5:0.5 *v*/*v*) and sonicated for 10 min in an ultrasonic bath (35 kHz, RK 100 SH, Bandelin Sonorex Super). Next, the solvent was separated by filtration, and the remaining solid was extracted two additional times with the same procedure and solvent system. The combined extracts were evaporated under reduced pressure to produce a slurry, which was subsequently dissolved in 50 mL of MeOH/H_2_O (1:1) and centrifuged for 10 min (7500 rpm). The supernatant liquid was extracted with petroleum ether (3 × 30 mL) to remove lipids and then concentrated under vacuum. The remaining residue was poured into 50 mL of brine and extracted repetitively with ethyl acetate (EtOAc, 3 × 50 mL) to remove the sugars, which were dissolved into the aqueous layer. The combined organic layers were dried over anhydrous MgSO_4_ and evaporated under reduced pressure to yield a solid, which was weighed and dissolved in MeOH to a concentration of 1 mg/mL and subjected to LC–MS/MS analysis.

Wine samples were processed immediately after bottle opening in accordance with the following procedure: 5 mL of wine were centrifuged for 10 min at 7500 rpm, membrane filtered (pore filter: 0.45μm) and subjected directly to LC–MS/MS analysis.

To avoid polyphenol degradation, all abovementioned activities were performed in the absence of direct sunlight and at temperatures below 35 °C.

### 2.4. Determination of Phenolic Compound Content by LC–MS/MS Analysis

#### 2.4.1. Preparation of Standard Stock Solutions

For each analyte (phenolic compound) standard, a methanolic stock solution was prepared at concentrations ranging from 90 to 2400 μg/mL. These solutions were maintained at −20 °C, and immediately prior to analysis, they were mixed in order to provide dilute solutions of mixtures of analytes at concentrations ranging from 50 to 1500 ng/mL. These solutions were further utilized to construct calibration curves.

#### 2.4.2. LC–MS/MS Analysis

The assessment of phenolic compounds was performed using an Accela Ultra High-Performance Liquid Chromatography system equipped with an autosampler and coupled with a TSQ Quantum Access triple-quadrupole mass spectrometer (Thermo Fisher Scientific, Inc., Waltham, MA, USA).

The separation of phytochemicals was achieved on a reverse phase column of internal diameter 150 × 2.1 mm and particle size 3 μm (Fortis Technologies Ltd., Neston, Cheshire, UK). For the protection of the column, an AF C18 guard column from the same provider was used with internal diameter 10 × 2.0 mm and particle size 3μm. Mobile phase A consisted of water/formic acid (0.1%) solution, while mobile phase B was composed of an acetonitrile/formic acid (0.1%) solution. Mobile phases A and B were degassed at 25 °C for 10 min. The injection volume of each sample was 10μL, the flow rate was set to 0.2 mL/min and the tray and column temperatures were set, respectively, at 25 and 35 °C. The gradient elution conditions for mobile phases A and B were set as follows: 0.0–2.0 min, 10% B; 2.0–16.7 min, from 10% to 100% B; 16.7–18.7 min, 100% B; and 18.8–22.0 min, 10% B for the re-equilibration of the column between injections.

For the MS/MS determination, the electrospray ionization (ESI) technique was utilized for both negative and positive ion polarities and operated in selected reaction monitoring (SRM) mode to achieve increased sensitivity. Before analysis, the molecular ion transitions and collision energies of the target analytes were obtained by direct infusion in full scan (mass range: 100–1500), while the ion source and vacuum parameters were optimized in order to be applicable for all analytes. A nitrogen generator (Peak Scientific) was used to provide the nitrogen that was utilized as sheath and auxiliary gases with the initial pressures set at 25 and 10 Arb, respectively. The spray voltage was set at 3.5 kV in the negative polarity and at 3.0 kV in the positive polarity. The capillary temperature was regulated at 300 °C, and the collision pressure of the argon gas was adjusted at 1.5 mTorr. All selected ion transitions are included in Table 1.

#### 2.4.3. Quantification of Phenolic Compounds

The quantification of phenolic compounds was achieved by diluting the corresponding standard stock solutions of target analytes in order to construct a calibration curve for each analyte for seven concentration levels (50, 100, 250, 500, 800, 1200 and 1500 ng/mL). The latter allowed for the quantification of the presence of the respective analytes by dividing the chromatographic peak area of the standard compound by the corresponding peak area of the internal standard (200 ng/mL) (A_analyte_/A_is_ = y_axis_). Next, the presence of each phenolic compound was defined by calculating its concentration, using the equation Conc. = x_axis_.

#### 2.4.4. Analytical Method Validation

##### Linearity

The linearity of the analytical method was evaluated using the aforementioned calibration curves in order to determine the respective correlation coefficients, slopes and intercept values. The linearity was considered acceptable when the regression coefficient exceeded 0.998 and the volume of the detected residuals remained lower than 20%.

###### Limit of Detection (LOD) and Limit of Quantification (LOQ)

The LOD and LOQ values were determined using the standard deviation of the response and slope, according to Equations (1) and (2), respectively:

(1) $$LOD=\frac{3.3\sigma }{S}\;\;$$

Equation (1): Calculation algorithm for the LOD (Limit of Detection) determination.

(2) $$LOQ=\frac{10\sigma }{S}\;\;$$

Equation (2): Algorithm for LOQ (Limit of Quantification) calculation.

Σ = standard deviation of response, S = slope of calibration curve.

The LOD and LOQ values, along with their respective equation and correlation coefficients, are presented in Table 2.

##### Precision

The precision of an analytical method is expressed as the relative standard deviation, %RSD of the repeatability (intra-day) and intermediate precisions (inter-day) of three analyses (n = 3) during the same day and over three days studied, respectively. Herein, the precision of the method was determined by analyzing the concentration of 250 ng/mL of the analyte standards using the aforementioned method of calculation. The precision was considered as acceptable when the %RSD value was lower of 20%.

##### Recovery

For the calculation of recoveries, three different amounts (low, medium and high) of the analyte standards were added to a white wine matrix. In the case of 15 analytes, which were determined to display low LOD and LOQ values, an additional (lower) amount of analyte standard was added. Next, each sample was treated according to its respective sample handling procedure and analyzed using the LC–MS/MS method. Finally, the quantity of each analyte was calculated using the corresponding calibration curve to provide the recovery value for each analyte.

##### Determination of Matrix Effect

The identification and quantification of an analyte can definitely be influenced by the matrix effect. This can lead to either a false negative output because of ion suppression or to a false positive result when the signal of the internal standard (IS) undergoes a suppression greater than that of the analyte.

The matrix effect can be quantitatively evaluated by comparing the response measured for a standard solution of analyte with the respective value obtained for a spiked solution of analyte at the same concentration and solvent with the analyzed samples. The matrix effect can be calculated by the following formula [23,39]:

(3) $$Matrix\;Effect\;\left(ME\right)=\frac{A_{StdS}-A_{Sample}}{A_{StdS}}\times 100\;$$

Equation (3): Algorithm for the calculation of the matrix effect.

A_StdS_ = peak area of analyte’s standard solution, A_Sample_ = peak area of spiked analyte in a sample not containing the analyte at the same concentration as the standard solution.

### 2.5. Estimation of Phenolic Compound Content and Antioxidant Properties

The estimation of the phenolic compounds abundances was achieved by the assessment of total phenolic content (TPC), total flavonoid content (TFC) and total tannin content (TTC). The measurement of antioxidant capacities was determined with 2,2-diphenyl-1-picrylhydrazyl (DPPH) and ferric reducing antioxidant power (FRAP) assays. The respective data were obtained using an Infinite^®^ 200 PRO microplate reader (Tecan Group Ltd., San Jose, CA, USA). All measurements were performed in triplicate, and the respective results were recorded as the mean ± standard deviation of the three replicates.

#### 2.5.1. Determination of Total Phenolic Content (TPC)

The TPCs for all samples were measured using a modified version of a spectrophotometric method developed by Hilma et al., 2018 [39]. Briefly, 10 μL of each sample was dissolved in 100 μL of water, and 10 μL of Folin–Ciocalteu reagent solution was added. The resulting solution was placed in triplicate in a 96-well microplate (Sarstedt AG & Co. KG, Nümbrecht, Germany) and incubated for 3 min at room temperature. Next, 20 µL of sodium carbonate aqueous solution (7.5% *w*/*v*) and 60μL of water were added, and the incubation was continued in the dark for an additional 60 min. The absorbance of the solution was measured at a 765 nm wavelength, and the respective sample was quantified relatively to a standard calibration curve prepared with 30–200 µg/mL (30, 55, 80, 110, 135, 160, 180, 200 µg/mL) solutions of gallic acid in methanol. The results are expressed as g of gallic acid equivalents per kg of dry weight of each sample (g GAE/kg of DW). For wines, the respective results are expressed as g of gallic acid equivalents per L of wine (g GAE/L of W).

#### 2.5.2. Determination of Total Flavonoid Content (TFC)

The TFCs of all samples were determined using a modification of the aluminum chloride method of Pękal and Pyrzynska [40]. Specifically, 100 µL of each sample, 50 µL of aluminum chloride aqueous solution (2% *w*/*v*) and 50 µL of sodium acetate aqueous solution (1 M) were poured in triplicate into a 96-well microplate and incubated in the dark at room temperature for 40 min. Next, the absorbance was measured at a 415 nm wavelength. The respective TFC values were calculated against a standard calibration curve prepared for quercetin solutions (10, 25, 40, 55, 70, 85, 100 µg/mL). Results are expressed as g of quercetin equivalents per kg of dry weight of each sample (g QE/kg DW) and for wines as g of quercetin equivalents per L of wine (g QE/L of W).

#### 2.5.3. Determination of Total Tannin Content (TTC)

The TTCs of all samples were estimated using a modified version of the method developed by Hong et al., 2021 [41]. In particular, 25 µL of all samples were mixed with 150 µL of a vanillin methanolic solution (4% *w*/*v*), and 25 µL of a methanolic solution of sulfuric acid (32%) were added. The mixture was placed in triplicate into a 96-well microplate and incubated for 15 min at room temperature in the dark. Next, the absorbance was measured at a 500 nm wavelength, and the TTCs were determined against a standard calibration curve of epicatechin solutions at concentrations of 120, 220, 350, 500, 650, 800, 950 and 1000 µg/mL. Results are expressed as g of epicatechin equivalents per kg of dry weight of each sample (g EE/kg DW) and for wines as g of epicatechin equivalents per L of wine (g EE/L of W).

#### 2.5.4. DPPH^∙^ Radical Scavenging Assay

The radical scavenging activity of the samples was evaluated using the DPPH^∙^ assay method modified by Anastasiadi et al., 2010 [35]. Briefly, 30 μL of each sample was mixed with 175 µL of a methanolic solution of DPPH radicals (0.1 M) and placed into a 96-well microplate. The mixture was incubated for 40 min at room temperature. Next, the absorbance was measured at a 515 nm wavelength, and the activity was determined against a standard curve generated for different concentrations of Trolox (15, 25, 35, 45, 55, 65, 75, 85 µg/mL). The percentage of inhibition (Pi) was calculated using the following equation:

(4) $$Pi=\left[1-\frac{A_{sample}-A_{blank}}{A_{control}-A_{blank}}\right]\times 100\;$$

Equation (4): percentage of inhibition.

A_sample_ = absorbance of sample, A_blank_ = absorbance of blank,

A_control_ = absorbance of control.

Results are expressed as the inhibitory concentrations (IC_50_) in g/kg dry weight for extracts or mg/L for wines that were required to quench 50% of the initial DPPH^∙^ radicals under the given experimental conditions.

#### 2.5.5. Ferric Reducing Antioxidant Power (FRAP) Assay

The determination of the reducing capacity of samples was based on a method published by Anastasiadi et al., 2010 [35], which concerned the determination of the samples’ ability to reduce the Fe^3+^ of a Fe^3+^-TPTZ complex (ferric-2,4,6-tripyridyl-s-triazine) into Fe^2+^-TPTZ. Specifically, the FRAP reagent was prepared just before the analysis by mixing 10 mL of acetate buffer (pH 3.6) with 1 mL of ferric chloride hexahydrate (20 mM in distilled water) and 1 mL of 2,4,6-tris(2-pyridyl)-s-triazine (TPTZ) (10 mM in HCl 0.04 N). The mixture was placed in a water bath adjusted to 37 °C. The mixture was allowed to reach 37 °C, 180 μL were pipetted into a 96-well microplate, and 30 μL of the sample were added. The microplate was incubated in the dark for 30 min at 37 °C, and then the absorbance was measured at a 593 nm wavelength and transformed to reducing capacity using a standard curve constructed for concentrations of FeSO_4_ ranging from 0 to 25 mM (5, 7, 9, 11, 15, 18, 21, 25 mM). The results are expressed in mol Fe^2+^/kg of dry weight for extracts and mol Fe^2+^/L for wines.

### 2.6. Statistical Analysis

The determination of peak identity was performed through correlation of the retention times with those of the reference standards. The analyte concentration was defined by integrating the peaks and creating standard calibration curves utilizing LCquan 2.7.0.20 software (Thermo Fisher Scientific, Inc., Waltham, MA, USA). Standard deviations for the LOD, LOQ assessments in triplicate were calculated using the statistical functions of Microsoft Office 365. One-way analysis of variance (ANOVA) was used to test whether there were significant differences between the mean values of different samples.

All results are presented as mean value ± standard deviation (SD) of experiments performed in triplicate. For all calculations performed in this work, the Durbin–Watson (DW) statistical tests for the residuals and the ANOVA table indicated that the P-value was always less than 0.05.

## 3. Results and Discussion

The antioxidant phenolic compounds represent a class of bioactive compounds that is responsible for the diverse health-beneficial properties of grapes, wines and vinification byproducts [2,3,4,7]. Thus, the development of an efficient method for the accurate quantitation of their presence is of paramount interest and the subject of numerous research endeavors [41,42,43].

The present study concerns the development and detailed validation of a versatile method that allows the simultaneous determination of a library containing 32 of the most common antioxidant phenolic compounds. The method developed was successfully applied to samples of grapes; vinification byproduct extracts; and red, rose and white wines. Furthermore, the antioxidant properties of extracts and wines were also evaluated.

### 3.1. Analytical Method Development

The novel analytical method refers to the simultaneous quantitation of the following antioxidant phytochemicals in various types of samples: 12 phenolic acids (gallic, protocatechuic, coutaric, fertaric, caftaric, *p*-coumaric, *o*-coumaric, chlorogenic, caffeic, syringic, ferulic, sinapic), 19 polyphenols (catechin, epicatechin, epigallocatechin, epigallocatechin gallate, epicatechin gallate, procyanidin A2, procyanidin B1, procyanidin B2, quercetin, myricetin, kaempferol, apigenin, hesperidin, isorhamnetin, *trans-*resveratrol, rutin, oenin, *trans-*polydatin, quercetin-3-*β*-D-glucoside) and coniferyl aldehyde, which is a phenolic compound extracted from cork stoppers into wine [44]. The method utilizes a LC–MS/MS triple quadrupole instrument and is suitable for the analysis of samples from grapes, wines and vinification byproducts. To date, the available analytical methods for the determination of these compounds mainly concern the simultaneous detection of only a limited number of compounds [45,46]. Thus, the efficient determination of their holistic phenolic profile still constitutes the subject of several research efforts.

A crucial feature for the novel method’s development was the definition of the suitable analytic conditions, which led to the efficient chromatographic separation of all target compounds, ensuring their good resolution in a short run time (not exceeding 22 min). In the few cases where an overlap between two compounds was observed, the application of selected reaction monitoring (SRM) transition for each analyte proved sufficient for the achievement of their proper identification and quantification. All analytes were detected by utilizing their respective deprotonated molecules as precursor ions in negative polarity [M-H]^−^ expect for oenin (3-glucoside of malvidin), which was detected as a protonated molecule in positive polarity [M+H]^+^.

### 3.2. Analytical Method Validation

The accreditation of the novel analytical method was performed through the determination of the following validation parameters: linearity, limit of detection (LOD), limit of quantification (LOQ), repeatability, precision, recovery and matrix effect.

#### 3.2.1. Determination of Linearity, LOD and LOQ

The linearity of the method was tested by injecting in triplicate, a mixture containing the 32 standard solutions of analytes plus an internal standard at seven concentration levels (50, 100, 250, 500, 800, 1200 and 1500 ng/mL). For each standard, a calibration curve was established, which was used for the determination of the respective coefficient (R^2^). The data obtained are included in Table 2 and are indicative of the good linearity observed for all analytes because in all cases the calculated R^2^ values ranged between 0.9988 and 0.9999.

The LOD and LOQ calculations were performed using Equations (1) and (2), respectively. The respective results verified the good sensitivity of the method because the obtained LOD values ranged from 7.5 to 158.3 ng/mL (Table 2).

#### 3.2.2. Repeatability, Recovery and Matrix Effect

The method repeatability was tested by injecting 3 replicate samples with 250 ng/mL concentration of each standard either during the same day (intra-day repeatability or run-to-run precision) or over the course of three days (inter-day repeatability or day-to-day precision). The intra- and inter-day repeatability was expressed as the relative standard deviation (RSD). The calculated RSDs ranged from 0.8 to 13.7% for run-to-run precision and from 0.9 to 14.4% for day-to-day precision, revealing a very good repeatability score for almost all cases.

The recovery for each standard was calculated by spiking each compound into a white wine sample for three quantity levels (low, medium and high). Next, the respective recoveries were determined by comparing the values obtained for each added compound in relation to the initial value of the compound contained in the sample. In particular, herein, the recoveries were determined for concentrations of 80, 300 and 1500 ng/mL except for analytes exhibiting LOD values < 50 ng/mL and LOQ < 100 ng/mL. For those compounds, the recovery calculation required the utilization of an additional (lower) concentration level (25 ng/mL). For all compounds, the calculated recovery values ranged between 74% and 120%, indicating the very good efficacy of the method (Table 3).

Finally, it must be pointed out that the matrix effect (ME) elimination is significant for the evaluation of an analytical method since is leading to a more realistic evaluation of the samples in a wide range of concentrations. The best-recognized technique available for ME correction refers to the internal standardization. In this study, the molecule of 2-(4-chlorophenyl) malonaldehyde was used as the internal standard, and the respective ME was calculated using Equation (2). It is evident that a ME value around 0% is indicative of ME absence. If ME is >0%, an ion suppression occurred, and for ME < 0%, an ion enhancement effect is observed. Herein, the calculated MEs for all analytes studied were <30% expect for epigallocatechin gallate and quercetin-3-*β* D-glucoside, which displayed, respectively, ME values of 35.5 and 34.8.

### 3.3. Estimation of TPC, TFC, TTC

The total contents of phenolics, flavonoids and tannins were determined for all studied samples through the assessment of their respective TPC, TFC and TTC scores. The results are summarized in Table 4, highlighting the red wine as displaying the highest TPC value (1.68 g of GAE/L), presumably because of the applied procedure of the red vinification technique. On the other hand, among the remaining samples, grape stems were determined to be the richest in phenolic compounds. This result is in line with literature reports [17,35] indicating that grape stems constitute a particularly rich source of antioxidant phenolics. In our case, it was noticeable that among the stems examined, those of the Monemvassia white variety were determined to be the richest in phenolic compounds, displaying a TPC value of 14.0 g GAE/kg. A similar trend with lower amounts was assessed for grape pomace samples with both the Monemvasia and Mandilaria varieties (white and red) exhibiting values of 4.49 and 5.10 g GAE/kg, respectively. Finally, grape samples were found to contain a lower concentration of phenolic compounds, presumably because the fresh crops of grapes contained a large number of additional biomaterials.

As expected, in all cases, the TFC values were lower as compared to the respective TPC values. It must be noted, however, that in this case, the stems of the Monemvassia variety were also determined to display the richest content (1.6 g QE/kg), while the remaining samples showed comparable values.

Finally, grapes, wines and pomaces of both red varieties studied displayed the richest tannin content (TTC), with stems exhibiting the highest tannin content. To our surprise, the stems of Monemvassia, a traditional, native Greek white variety, displayed the highest value amongst all tested samples (64 g EE/kg). This finding is indicative of the difference in tannin content between the woody part of the plant and its crops and products. In addition, it demonstrates the great potential for this variety’s vinification byproducts (pomace and stems) to function as potent candidates for commercial exploitation toward the isolation of extracts enriched with antioxidant phenols to be used for the production of cosmetics and food supplements.

### 3.4. Phenolic Compound Quantitation

The identification of phenolic compounds was based on their mass-to-charge (*m*/*z*) values in both negative and positive ionization modes, while their quantitation was achieved utilizing the calibration curves constructed with the respective standard compounds. The respective results are presented in Table 5, Table 6 and Table 7. The concentration of each phenolic compound is expressed as mg/kg of dry matter for vinification byproducts and lyophilized grapes and as mg/L for the wine samples.

**Figure 1:**
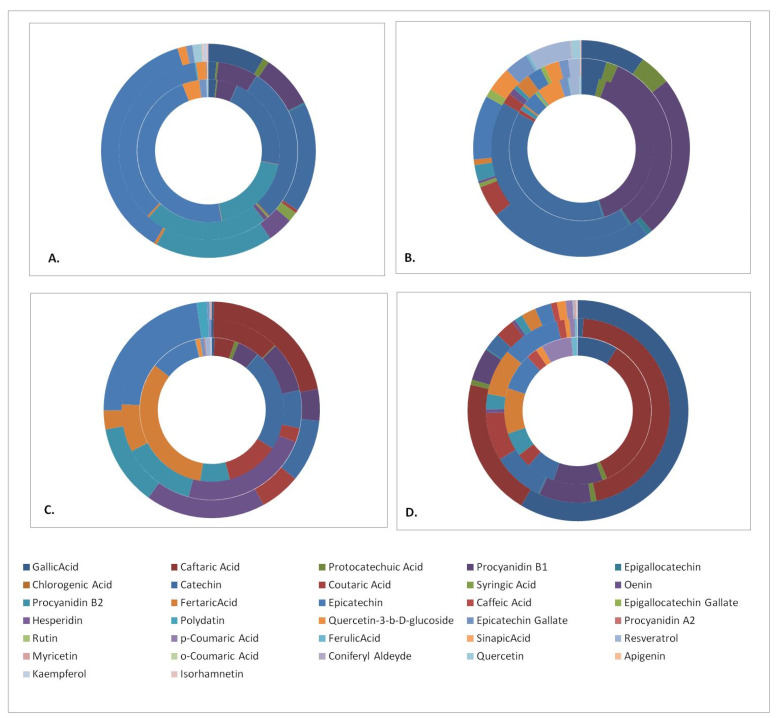
Phenolic compound content of samples studied. (**A**) pomace, (**B**) stems, (**C**) grapes and (**D**) wines. Varieties: inner disk, Monemvassia; middle disk, Mandilaria; outer disk, Aidani mavro.

In order to facilitate the outcome comparisons, the polyphenols quantitation results were cumulated in disk diagrams (Figure 1). Each disk is composed of an outer part representing the contents of the Aidani mavro variety, a middle part for Mandilaria and an inner part for the Monemvassia variety. There are four disks representing, respectively, the phenolic contents of the pomace, stems, grapes and wines of the investigated varieties. In general, the results indicated that pomaces and stems displayed the richest polyphenolic content, which was composed of a variety of phenolic acids and flavonol monomers, dimers and glycosides.

#### 3.4.1. Grape Berries

The respective results (Table 5, Table 6 and Table 7) are indicative of the rich diversity of the detected phenolic constituents in all grapevines tested, highlighting the phenolic compound content of Monemvassia, a white variety, as comparable to those of the red varieties. The most notable difference among the phenolic profiles of the three varieties referred to their oenin (malvidine-3-glycoside) content, which was exclusively detected in berries and wines of red varieties. In respect to the presence of the remaining phenolic compounds, it was evident that their bulk in all samples tested was composed of phenolic acids, especially fertaric, coutaric and caftaric acids, which were assayed in concentrations ranging, respectively, from 154.0 to 22.5, 56 to 23.9 and 171 to 21.4 mg/kg of dry material. Several other acids were determined in lower concentrations. These results are indicative of the acidic character of grapevine varieties native to Greek islands. The remaining bulk of the phenolic compounds consisted of flavan-3-ol monomers and their glycosides, with prevailing compounds monomers (+)-catechin and (–)-epicatechin, which were found in concentrations ranging, respectively, from 107 to 19 and 181 to 50 mg/kg of dry material, while only traces of the remaining flavonol monomers were detected. These results correlate well with previous findings for grapevines from the Greek islands [47]. On the contrary, only low amounts of flavonol glycosides were detected with the most abundant being epicatechin gallate and quercetin-3-*β*-glucoside. In respect to the flavonol dimers, the presence of large amounts of procyanidines B1 and B2 was detected in concentrations ranging from 37.8 to 23 and 96 to 31 mg/kg dry material, respectively. Finally, the presence of bioactive stilbene *trans-*resveratrol was detected in the white variety, while its glycoside, *trans*-polydatin, was present in large amounts in both red varieties tested.

#### 3.4.2. Grape Pomace

The direct comparison of the phenolic compound profiles detected in grape pomaces (Table 5, Table 6 and Table 7) with those in grape berries revealed a series of significant differences in respect to their qualitative and quantitative profiles. The investigated pomaces were particularly rich in the monomeric flavon-3-ols (+)-catechin and (–)-epicatechin in concentrations exceeding 1000 mg/kg. In addition, the presence of substantial amounts of flavonol glycosides such as quercetin-3-*β*-glucoside and epicatechin gallate was also revealed. Pomace samples were also determined as particularly rich in the flavonol dimers procyanidin B1 and B2 at concentrations ranging, respectively, from 266 to 66 and 146 to 760 mg/kg of dry material. On the contrary, their content of phenolic acids was limited to only small amounts. Finally, the concentration of stilbenes was significantly low because these phytoalexins were particularly susceptible to degradation.

#### 3.4.3. Grape Stems

Grape stems, the woody part of the grapevine, were found to display the highest TPC values (Table 3). The quantitation of their polyphenolic compound content (Table 5, Table 6 and Table 7) determined as the prevailing molecules the flavon-3-ol monomer (+)-catechin in concentrations ranging from 1176 to 127 mg/kg of dry material and the flavonol dimer procyanidin B1 (942 to 125 mg/kg of DM). The presence of various phenolic acids and flavonol dimers was also detected in stems. The most characteristic finding refers to their particularly rich content of *trans-*resveratrol, a very potent bioactive phenolic compound [17,18,19] with very high added and market values. This is indicative of the great potential for grape stems for use as a source for the production of bioactive extracts or molecules.

#### 3.4.4. Wines

The great plethora of reactions involving phenolic compounds, carried out during the winemaking and maturation processes (reactions of chemical and enzymatic oxidations, condensation, hydrolysis, etc.) [47], results in the occurrence in wines of a complex profile of diverse phenolic compounds. As was expected, red wine (Mandilaria) displayed the richest and most diversified content of phenolic compounds, presumably because of the variety [35] and the red vinification technique. The phenolic acid profile of this wine was composed of a broader variety of molecules as compared to those of grape berries. Prevailing compounds were gallic, caftaric, fertaric and coutaric acids (Table 5) with gallic acid being the most abundant. The presence of several flavonol monomers, dimers and glycosides was also revealed in smaller quantities. It is noteworthy, however, that Monemvassia, a traditional native Greek variety, which was studied herein for the first time, produced a white wine that was quite rich in phenolic compounds.

#### 3.4.5. Stilbene Content

Stilbenes, represented by *trans*-resveratrol and *trans*-polydatin (known also as *trans*-piceid), constitute the most prominent bioactive phytoalexins. They consist of two phenol rings linked to each other by an ethylene bridge, and their presence in various grapevine varieties and related derivatives is well-established. During the last decades, several studies have verified the diverse bioactivities of *trans-*resveratrol, emphasizing its particularly potent antioxidant [17,19] and anticancer activities [18,19] along with numerous health benefits including anti-inflammatory, cardioprotective, vasorelaxant, phytoestrogenic and neuroprotective effects [48]. Accordingly, *trans-*polydatin, the 3-*β*-mono-D-glucoside glycoside (the natural precursor of *trans-*resveratrol), is also known for its prominent antioxidant and antiproliferative activities [49].

**Figure 2:**
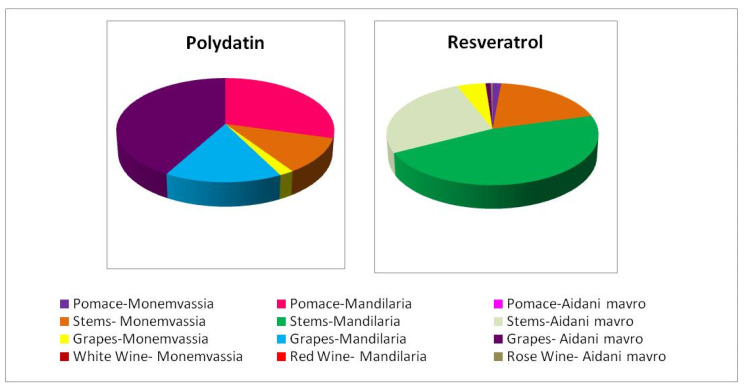
Distribution of *trans-*resveratrol and *trans-*polydatin content in samples studied.

Figure 2 shows the *trans-*polydatin and *trans-*resveratrol content of the samples studied. It was evident that samples containing *trans-*resveratrol were particularly poor in *trans-*polydatin and vice versa except Monemvassia stems and grapes and Aidani mavro grapes, which contained both stilbenes. It must be noted that in all cases, stems were found to contain the highest concentration of *trans*-resveratrol, indicating their great potential to act as a rich source of this bioactive molecule.

### 3.5. Antioxidant Activity (DPPH^∙^ and FRAP Assays)

The antioxidant activities of samples tested were determined through DPPH^∙^ and FRAP assays. The respective results are included in Table 8. The results for the DPPH^∙^ assay, which are expressed as IC_50_ values, determined that the stems of the Monemvassia variety were substantially the most potent among the samples investigated. This result is perfectly in line with all findings, which have indicated that stems of this variety displayed the richest content of phenolic compounds and the higher TPC values. Regarding the wine samples, those originated from red grape varieties (red and rose wines) exhibited significantly more potent antioxidant capacities.

Accordingly, similar results were observed for the determination of the reducing capacities of the samples studied, which were estimated with the FRAP assay and highlighted the stems of the Monemvassia variety as the most active among all samples. Finally, among the wines studied, the red wine, Paros Reserve, was by far the most potent.

## 4. Conclusions

A novel method for the fast and accurate quantitation of the 32 most common antioxidant phenolic compounds present in grapevines was developed and fully validated on LC–MS/MS triple quadrupole. The method was successfully applied for wine, grape berries, pomace and stems sampled from three native Greek *Vitis vinifera* species. Results indicated that the stems of the Monemvassia white variety are particularly rich in high-added-value phenolic compounds such as (+)-catechin, procyanidin B1 and *trans-*resveratrol. This finding is in line with their TPC, TFC and TTC contents and the determination of their antioxidant capacities with DPPH^∙^ and FRAP assays. Similar results were obtained for white wine produced from the same variety.

The analytical data herein are the first reported in the literature for the varieties of the Monemvassia and Aidani mavro grapevines and are indicative that stems of the white Greek native grapevine Monemvassia possess great ptoentials to serve as rich sources of high-added-value antioxidant phenolic compounds.

## Figures and Tables

**Table 1:** Transition, collision energy, polarity and retention time (RT) applied for each phenolic standard.

Compound	Parent Mass	Product Mass	Collision Energy (eV)	Polarity	RT (min)
Gallic acid	169.939	126.089	17	(–)	3.59
		125.047	17		
Protocatechuic acid	154.057	109.048	22	(–)	6.43
		110.095	17		
Caftaric acid	312.151	149.039	14	(–)	6.82
		179.985	17		
Procyanidin B1	578.328	426.099	18	(–)	6.89
Epigallocatechin	306.138	124.855	27	(–)	7.13
		179.658	18		
Chlorogenic acid	354.200	191.113	20	(–)	7.72
Oenin	493.236	315.121	47	(+)	7.87
		331.122	23		
Catechin	290.133	203.873	22	(–)	7.89
		245.958	17		
Procyanidin B2	578.122	290.047	31	(–)	8.06
Coutaric acid	296.129	120.145	29	(–)	8.16
		164.015	18		
Fertaric acid	326.172	134.113	33	(–)	8.46
		194.059	18		
Epicatechin	290.132	203.818	21	(–)	8.48
		245.948	17		
Epigallocatechin gallate	458.233	167.890	22	(–)	8.59
		457.460	11		
Caffeic acid	180.102	135.095	24	(–)	8.69
		136.106	19		
Syringic acid	198.085	167.89	22	(–)	8.86
		182.921	16		
Hesperidin	610.054	301.320	28	(–)	8.96
*trans-*Polydatin	390.548	229.992	21	(–)	9.31
		389.777	8		
Quercetin-3-*β*-D-glucoside	464.220	300.781	28	(–)	9.32
		301.966	26		
Epicatechin gallate	442.252	168.845	23	(–)	9.47
		290.236	21		
Procyanidin A2	576.358	424.052	18	(–)	9.59
		449.815	24		
Rutin	610.355	271.536	68	(–)	9.65
		302.205	44		
*p*-Coumaric acid	164.014	94.475	36	(–)	9.85
		119.835	18		
Sinapic acid	224.132	193.987	24	(–)	9.99
		209.043	17		
Ferulic acid	194.120	135.094	20	(–)	10.09
		179.062	16		
Myricetin	318.114	136.79	29	(–)	10.63
		178.963	22		
*o*-Coumaric acid	163.970	119.068	17	(–)	10.79
		120.127	16		
Coniferyl aldeyde	178.085	162.944	16	(–)	11.10
*trans*-Resveratrol	228.146	144.131	29	(–)	11.24
		186.109	22		
Quercetin	302.111	151.483	24	(–)	11.74
		179.692	22		
Apigenin	270.037	116.922	42	(–)	12.67
		117.972	40		
Kaempferol	286.102	211.942	33	(–)	12.83
		229.944	27		
Isorhamnetin	316.376	301.277	24	(–)	13.01
		302.404	23		
*Internal Standard*: 2-(4-Chlorophenyl) malonaldehyde	182.456	136.900	26	(–)	12.60
		154.892	19		

**Table 2:** Calculations of equations, correlation coefficients, LOD and LOQ for each phenolic standard.

Compound	Equation	R^2^	LOD (ng/mL)	LOQ (ng/mL)
Gallic acid	y = −0.00096256 + 0.0395411x	0.9998	29.2	88.4
Caftaric acid	y = −0.00534158 + 0.07718x	0.9996	25.3	76.8
Protocatechuic acid	y = −0.000761486 + 0.0310679x	0.9999	29.9	90.8
Procyanidin B1	y = −0.00258365 + 0.0256469x	0.9998	53.2	161.2
Epigallocatechin	y = −0.00488722 + 0.0509023x	0.9997	63.4	192.1
Chlorogenic acid	y = −0.00016251 + 0.0821949x	0.9992	25.4	76.9
Catechin	y = −0.00202138 + 0.0129254x	0.9993	94.4	286.1
Coutaric acid	y = −0.007455 + 0.198158x	0.9998	49.0	148.4
Syringic acid	y = −0.00125888 + 0.0655096x	0.9998	119.7	362.8
Oenin	y = −0.406027 + 11.2312x	0.9995	20.5	62.1
Procyanidin B2	y = −0.00111717 + 0.0215283x	0.9996	62.2	188.6
Fertaric acid	y = 0.000358572 + 0.147193x	0.9999	7.5	22.6
Epicatechin	y = 0.0000486191 + 0.00498918x	0.9989	46.2	139.6
Caffeic acid	y = −0.167715 + 0.488001x	0.9998	11.9	36.1
Epigallocatechin gallate	y = 0.000118646 + 0.00994617x	0.9993	34.5	104.5
Hesperidin	y = 0.000321812 + 0.000883822x	0.9988	120.0	363.6
*trans*-Polydatin	y = 0.00124534 + 0.0569565x	0.9998	59.9	181.4
Quercetin-3-*β*-D-glucoside	y = −0.000728175 + 0.0484013x	0.9998	41.6	126.1
Epicatechin gallate	y = −0.00151726 + 0.0801815x	0.9999	32.5	98.5
Procyanidin A2	y = −0.000351333 + 0.131369x	0.9998	46.3	140.4
Rutin	y = −0.00209729 + 0.0613716x	0.9997	42.9	130.1
*p*-Coumaric acid	y = −0.000916311 + 0.0643206x	0.9998	30.4	92.4
Ferulic acid	y = −0.00093805 + 0.01891x	0.9994	81.1	245.7
Sinapic acid	y = 0.000677742 + 0.00960727x	0.9996	53.9	163.4
*trans-*Resveratrol	y = 0.000015519 + 0.00578912x	0.9999	158.3	479.8
Myricetin	y = 0.000640023 + 0.20355x	0.9998	13.0	39.5
*o*-Coumaric acid	y = −0.0000876583 + 0.0740954x	0.9997	28.9	87.7
Coniferyl aldehyde	y = −0.00119489 + 0.0676759x	0.9999	31.3	95.0
Quercetin	y = −0.00140527 + 0.068703x	0.9999	31.3	95.0
Apigenin	y = 0.00884981 + 0.300563x	0.9993	8.3	25.2
Kaempferol	y = −0.000290336 + 0.0430157x	0.9995	52.2	158.2
Isorhamnetin	y = −0.00579894 + 0.432144x	0.9999	15.7	47.5

**Table 3:** Calculation of recovery, precision and ME (matrix effect) for each phenolic standard.

Compound	Recoveries % (±RSD) (n = 3)				Intra-Day Precision % (±RSD)	Inter-Day Precision % (±RSD)	ME (%)
	25 ng/mL	80 ng/mL	300 ng/mL	1500 ng/mL	250 ng/mL	250 ng/mL	
Gallic acid	104.6–123.0	69.0–82.4	115.9–127.9	95.9–104.5	2.2	4.5	−20.4
Caftaric acid	104.8–107.6	100.2–105.4	92.6–104.0	94.7–98.5	3.7	11.8	18.0
Protocatechuic acid	95.1–102.9	98.8–101.6	111.9–117.5	100.4–106.0	13.7	8.0	−11.3
Procyanidin B1	-	92.5–104.7	104.4–109.4	79.5–92.3	5.6	0.9	−5.4
Epigallocatechin	-	93.6–96.2	92.9–104.3	97.8–103.0	13.3	3.4	−21.0
Chlorogenic acid	79.5–83.7	62.0–76.0	73.4–78.8	76.5–79.9	8.6	14.4	4.3
Catechin	-	72.3–75.1	76.9–82.5	79.6–83.4	4.9	2.9	−0.6
Coutaric acid	-	89.4–95.0	89.4–95.0	75.9–91.7	3.9	8.2	3.1
Syringic acid	-	91.6–103.4	99.9–108.3	109.6–116.0	9.5	1.1	28.0
Oenin	97.0–101.8	118.4–122.0	102.9–103.7	97.5–102.3	4.8	9.4	−22.0
Procyanidin B2	-	80.4–80.8	84.1–89.7	80.2–89.8	6.1	5.2	−5.7
Fertaric acid	91.0–108.2	92.5–104.7	100.2–105.4	94.9–98.5	6.1	5.0	12.9
Epicatechin	-	84.1–90.3	78.8–80.8	87.8–100.4	10.0	7.0	10.5
Caffeic acid	113.8–121.9	96.7–99.5	107.8–112.0	115.7–117.3	5.3	3.5	19.2
Epigallocatechin gallate	-	76.3–77.3	87.4–91.2	96.2–97.4	7.7	3.6	35.5
Hesperidin	-	79.0–84.4	72.2–75.8	65.1–77.9	7.9	7.4	8.7
*trans*-Polydatin	-	84.0–88.0	98.0–107.0	84.6–85.4	3.9	5.0	9.7
Quercetin-3-*β*-D-glucoside	-	96.4–112.0	92.9–104.5	101.1–112.7	6.5	5.4	34.8
Epicatechin gallate	94.9–115.7	73.4–81.8	69.1–82.7	69.8–82.6	4.0	2.9	18.9
Procyanidin A2	-	98.0–101.8	75.3–80.9	85.0–87.0	11.5	9.7	−0.5
Rutin	-	71.2–85.0	71.9–79.1	71.2–85.0	2.9	2.3	24.3
*p*-Coumaric acid	103.2–121.6	69.6–74.8	83.8–85.2	113.7–115.7	4.3	5.2	15.2
Ferulic acid	-	98.2–104.0	77.2–79.8	77.8–81.8	8.0	12.3	1.2
Sinapic acid	-	104.6–110.4	65.6–81.2	78.8–83.2	7.9	11.2	2.8
*trans-*Resveratrol	-	107.2–111.4	90.7–105.3	66.7–74.7	1.9	1.7	27.2
Myricetin	69.7–78.5	89.3–97.7	97.5–10.5	84.8–88.0	3.4	3.1	−19.9
*o*-Coumaric acid	79.2–84.4	89.6–110.0	82.0–101.4	78.1–84.7	3.9	1.7	−1.2
Coniferyl aldehyde	82.1–101.7	66.7–73.5	79.8–93.8	65.1–75.5	0.8	4.5	4.7
Quercetin	94.8–105.2	70.5–74.9	75.2–88.4	75.0–75.2	2.4	6.2	2.3
Apigenin	74.0–77.4	77.6–82.6	83.9±85.9	81.4–84.6	7.3	4.0	−14.8
Kaempferol	-	70.3–73.6	73.1–76.5	87.7–90.3	1.3	2.0	−11.5
Isorhamnetin	102.9–106.1	88.0–94.4	83.2–87.8	75.2–77.6	6.4	5.4	0.4

**Table 4:** Total phenolic (TPC), flavonoid (TFC) and tannin (TTC) contents of samples tested.

Grape Variety	Color	Part	TPC (g GAE/kg)	TFC (g QE/kg)	TTC (g EE/kg)
Mandilaria	Red	Grape	0.18 ± 0.02	0.22 ± 0.01	12.3 ± 0.5
		Pomace	5.1 ± 0.1	0.20 ± 0.02	15.6 ± 0.4
		Stem	4.95 ± 0.08	0.299 ± 0.006	18.2 ± 0.8
Monemvassia	White	Grape	0.287 ± 0.006	0.167 ± 0.004	0.53 ± 0.08
		Pomace	4.49 ± 0.08	0.22 ± 0.02	12 ± 1
		Stem	14.0 ± 0.3	1.6 ± 0.1	64 ± 2
Aidani mavro	Red	Grape	0.94 ± 0.03	0.32 ± 0.01	4.6 ± 0.1
		Pomace	0.25 ± 0.01	0.17 ± 0.02	9.5 ± 0.4
		Stem	2.46 ± 0.02	0.274 ± 0.009	8.3 ± 0.4
**Wine Appellation**	**Color**	**Grape Variety**	**TPC** **(g GAE/L)**	**TFC** **(g QE/L)**	**TTC** **(g EE/L)**
Paros Reserve	Red	Mandilaria	1.68 ± 0.02	0.127 ± 0.004	3.8 ± 0.1
Amphora	White	Monemvassia	0.30 ± 0.04	0.0227 ± 0.0007	0.56 ± 0.05
Rose Moraitis	Rose	Aidani mavro	0.22 ± 0.02	0.0175 ± 0.0004	0.38 ± 0.03

Data are reported on a dry weight basis. GAE: gallic acid equivalent; QE: quercetin equivalent; EE: epicatechin equivalent.

**Table 5:** Quantitation of phenolic compound content in the Mandilaria red variety (mg/kg DW) and wine (mg/L).

Compound	Grapes	Wine	Pomace	Stems
Gallic acid	2.5 ± 0.1	180 ± 7	54 ± 2	125 ± 8
Caftaric acid	171 ± 2	62 ± 1	ND	ND
Protocatechuic acid	ND	2.32 ± 0.10	13 ± 1	59 ± 8
Procyanidin B1	37.8 ± 0.5	14.7 ± 0.4	266 ± 3	942 ± 19
Epigallocatechin	tr	0.55 ± 0.07	1.8 ± 0.1	8.9 ± 0.4
Chlorogenic acid	ND	ND	ND	ND
(+)-Catechin	74 ± 3	7.8 ± 0.4	1049 ± 16	1176 ± 43
Coutaric acid	48 ± 2	8.66 ± 0.08	5.20 ± 0.01	54 ± 2
Syringic acid	ND	ND	12 ± 3	ND
Oenin	142 ± 3	1.5 ± 0.04	28 ± 3	33 ± 9
Procyanidin B2	96 ± 4	3.7 ± 0.2	817 ± 10	21 ± 1
Fertaric acid	22.5 ± 0.9	6.6 ± 0.2	13.00 ± 0.05	72.9 ± 0.2
(–)-Epicatechin	181 ± 8	7.2 ± 0.3	1299 ± 7	71 ± 5
Caffeic acid	ND	2.77 ± 0.04	ND	ND
Epigallocatechin gallate	ND	ND	tr	22.0 ± 1.0
Hesperidin	ND	ND	ND	ND
*trans-*Polydatin	12 ± 3	ND	8.41 ± 0.01	ND
Quercetin-3-*β*-D-glucoside	ND	3.94 ± 0.09	66 ± 4	73 ± 3
Epicatechin gallate	3.0 ± 0.1	0.022 ± 0.006	4.4 ± 0.1	46 ± 3
Procyanidin A2	tr	ND	ND	ND
Rutin	0.052 ± 0.001	ND	ND	ND
*p*-Coumaric acid	ND	3.05 ± 0.07	tr	1.2 ± 0.2
Ferulic acid	ND	0.22 ± 0.05	ND	tr
Sinapic acid	tr	ND	ND	ND
*trans-*Resveratrol	1.1 ± 0.3	0.37 ± 0.08	tr	58 ± 4
Myricetin	1.76 ± 0.04	0.87 ± 0.03	0.92 ± 0.03	0.98 ± 0.01
*o*-Coumaric acid	ND	ND	ND	ND
Coniferyl aldehyde	ND	ND	ND	ND
Quercetin	ND	ND	ND	ND
Apigenin	ND	ND	6.0 ± 0.3	3.0 ± 0.2
Kaempferol	ND	0.374 ± 0.009	ND	ND
Isorhamnetin	tr	0.60 ± 0.01	2.29 ± 0.02	1.77 ± 0.05

Data from the extracted samples are reported on a dry weight basis. ND: not determined; tr: trace. Data are presented as mean ± standard deviation of three replicates.

**Table 6:** Quantitation of phenolic compound content in the Aidani mavro red variety (mg/kg DW) and wine (mg/L).

Compound	Grapes	Wine	Pomace	Stems
Gallic acid	1.06 ± 0.2	0.65 ± 0.02	70 ± 3	49 ± 2
Caftaric acid	33.5 ± 0.5	30.9 ± 0.6	ND	ND
Protocatechuic acid	0.47 ± 0.07	0.64 ± 0.04	7.9 ± 0.5	24.3 ± 0.8
Procyanidin B1	25.1 ± 0.2	6.2 ± 0.2	66 ± 2	125 ± 7
Epigallocatechin	tr	0.12 ± 0.03	1.8 ± 0.1	4.3 ± 0.3
Chlorogenic acid	ND	ND	ND	ND
(+)-Catechin	19 ± 1	6.1 ± 0.4	136 ± 6	127 ± 5
Coutaric acid	6.97 ± 0.09	5.7 ± 0.08	3.8 ± 0.3	24.1 ± 0.7
Syringic acid	ND	ND	12 ± 3	3.0 ± 0.5
Oenin	65 ± 1	0.446 ± 0.009	32 ± 2	1.9 ± 0.2
Procyanidin B2	37 ± 2	1.8 ± 0.2	146 ± 5	12.3 ± 0.6
Fertaric acid	23.9 ± 0.6	5.5 ± 0.2	3.6 ± 0.1	4.3 ± 0.3
(–)-Epicatechin	61 ± 4	6.9 ± 0.9	302 ± 14	49 ± 2
Caffeic acid	ND	0.92 ± 0.02	ND	ND
Epigallocatechin gallate	ND	ND	ND	6.4 ± 0.3
Hesperidin	ND	ND	ND	ND
*trans-*Polydatin	4.46 ± 0.03	ND	ND	ND
Quercetin-3-*β*-D-glucoside	ND	0.56 ± 0.02	10.6 ± 0.3	18.8 ± 0.4
Epicatechin gallate	1.7 ± 0.2	ND	7.9 ± 0.4	18.4 ± 0.5
Procyanidin A2	tr	tr	ND	ND
Rutin	tr	ND	ND	ND
*p*-Coumaric acid	ND	0.64 ± 0.04	ND	ND
Ferulic acid	ND	0.25 ± 0.04	ND	1.9 ± 0.2
Sinapic acid	ND	ND	ND	ND
*trans-*Resveratrol	tr	tr	ND	33 ± 3
Myricetin	tr	ND	ND	0.45 ± 0.01
*o*-Coumaric acid	ND	ND	ND	ND
Coniferyl aldehyde	ND	ND	ND	ND
Quercetin	ND	ND	11.5 ± 0.4	7.2 ± 0.3
Apigenin	ND	ND	1.5 ± 0.1	tr
Kaempferol	ND	0.067 ± 0.007	4.4 ± 0.4	tr
Isorhamnetin	ND	0.072 ± 0.002	2.4 ± 0.1	0.99 ± 0.01

Data from the extracted samples are reported on a dry weight basis. ND: not determined; tr: trace. Data are presented as mean ± standard deviation of three replicates.

**Table 7:** Quantitation of phenolic compound content in the Monemvassia white variety (mg/kg DW) and wine (mg/L).

Compound	Grapes	Wine	Pomace	Stems
Gallic acid	2.77 ± 0.03	9 ± 1	72 ± 5	40.3 ± 0.6
Caftaric acid	21.4 ± 0.3	35 ± 1	ND	ND
Protocatechuic acid	4.2 ± 0.1	1.04 ± 0.05	11 ± 1	16.8 ± 0.6
Procyanidin B1	23 ± 2	11.0 ± 0.3	181 ± 7	400 ± 2
Epigallocatechin	0.47 ± 0.01	tr	2.9 ± 0.1	3.08 ± 0.08
Chlorogenic acid	ND	ND	tr	ND
(+)-Catechin	107 ± 9	6.4 ± 0.9	866 ± 8	387 ± 5
Coutaric acid	56 ± 3	3.11 ± 0.05	3.65 ± 0.03	8.67 ± 0.08
Syringic acid	ND	ND	ND	ND
Oenin	0.12 ± 0.01	ND	1.06 ± 0.01	0.57 ± 0.01
Procyanidin B2	31 ± 1	5.3 ± 0.2	760 ± 13	10.4 ± 0.4
Fertaric acid	154 ± 8	10.1 ± 0.1	4.4 ± 0.5	4.4 ± 0.2
(–)-Epicatechin	50 ± 2	8.2 ± 0.5	1901 ± 68	34 ± 1
Caffeic acid	ND	2.4 ± 0.1	ND	ND
Epigallocatechin gallate	ND	ND	tr	5.51 ± 0.08
Hesperidin	ND	ND	ND	ND
*trans-*Polydatin	0.55 ± 0.08	ND	ND	3.2 ± 0.2
Quercetin-3-*β*-D-glucoside	4.9 ± 0.3	1.56 ± 0.06	155 ± 3	54.3 ± 0.6
Epicatechin gallate	3.9 ± 0.2	ND	67 ± 2	19.0 ± 0.3
Procyanidin A2	1.0 ± 0.1	ND	ND	ND
Rutin	tr	ND	ND	ND
*p*-Coumaric acid	ND	6.7 ± 0.4	ND	ND
Ferulic acid	tr	1.3 ± 0.2	1.1 ± 0.1	2.5 ± 0.2
Sinapic acid	ND	ND	ND	ND
*trans-*Resveratrol	6.0 ± 0.8	ND	1.8 ± 0.8	24.0 ± 0.9
Myricetin	ND	ND	ND	ND
*o*-Coumaric acid	ND	ND	ND	ND
Coniferyl aldehyde	ND	ND	ND	ND
Quercetin	ND	ND	13 ± 2	5.2 ± 0.2
Apigenin	ND	ND	tr	tr
Kaempferol	1.3 ± 0.1	0.097 ± 0.007	1.4 ± 0.1	ND
Isorhamnetin	tr	0.027 ± 0.001	1.2 ± 0.3	ND

Data from the extracted samples are reported on a dry weight basis. ND: not determined; tr: trace. Data are presented as mean ± standard deviation of three replicates.

**Table 8:** Results of DPPH and FRAP assays for the determination of antioxidant capacities of the investigated samples.

Grape Variety	Color	Part	DPPH IC_50_g TΕ/kg DW	FRAP mol E-Fe(II)/kg DW
Mandilaria	Red	Grapes	40.3 ± 0.3	0.12 ± 0.04
		Pomace	30.2 ± 0.2	0.31 ± 0.05
		Stems	24.20 ± 0.04	0.35 ± 0.07
Monemvassia	White	Grapes	69.6 ± 1.2	0.03 ± 0.01
		Pomace	30.1 ± 0.1	0.32 ± 0.09
		Stems	5.634 ± 0.009	1.4 ± 0.4
Aidani mavro	Red	Grapes	67.4 ± 0.2	0.06 ± 0.02
		Pomace	33.31 ± 0.07	0.21 ± 0.05
		Stems	57.2 ± 0.1	0.18 ± 0.04
**Wine Appellation**	**Color**	**Grape Variety**	**IC_50_ (mg TE/L)**	**mol E-Fe(II)/L**
Paros Reserve	Red	Mandilaria	0.047 ± 0.002	0.031 ± 0.003
Amphora	White	Monemvassia	0.121 ± 0.001	0.010 ± 0.002
Rose Moraitis	Rose	Aidani mavro	0.088 ± 0.002	0.014 ± 0.002

TE: Trolox equivalents; E-Fe(II): iron (II) equivalents. Data from the extracted samples are reported on a dry weight basis and presented as mean ± standard deviation of three replicates.

## Data Availability

Data is contained within the article.
